# The interplay between immune maturation, age, chronic viral infection and environment

**DOI:** 10.1186/s12979-015-0030-3

**Published:** 2015-05-09

**Authors:** Kristie L Oxford, Myra Grace A dela Pena-Ponce, Kara Jensen, Meghan K Eberhardt, Abigail Spinner, Koen KA Van Rompay, Joseph Rigdon, Katie R Mollan, VV Krishnan, Michael G Hudgens, Peter A Barry, Kristina De Paris

**Affiliations:** Center of Comparative Medicine, University of California, Davis, California USA; Department of Microbiology and Immunology, University of North Carolina, Burnett-Womack Bldg, 160 Dental Circle, Chapel Hill, NC 27599-7292 USA; California National Primate Research Center, University of California, Davis, California USA; Gillings School of Public Health, University of North Carolina, Chapel Hill, North Carolina USA; Center for AIDS Research, University of North Carolina, Chapel Hill, North Carolina USA; Department of Pathology and Laboratory Medicine, University of California, Davis, California USA

**Keywords:** Aging, Immune maturation and function, Rhesus CMV infection, Rhesus macaques, Immune development and maturation, RhCMV infection, Aging, Inflammaging

## Abstract

**Background:**

The worldwide increase in life expectancy has been associated with an increase in age-related morbidities. The underlying mechanisms resulting in immunosenescence are only incompletely understood. Chronic viral infections, in particular infection with human cytomegalovirus (HCMV), have been suggested as a main driver in immunosenescence. Here, we propose that rhesus macaques could serve as a relevant model to define the impact of chronic viral infections on host immunity in the aging host. We evaluated whether chronic rhesus CMV (RhCMV) infection, similar to HCMV infection in humans, would modulate normal immunological changes in the aging individual by taking advantage of the unique resource of rhesus macaques that were bred and raised to be Specific Pathogen Free (SPF-2) for distinct viruses.

**Results:**

Our results demonstrate that normal age-related immunological changes in frequencies, activation, maturation, and function of peripheral blood cell lymphocytes in humans occur in a similar manner over the lifespan of rhesus macaques. The comparative analysis of age-matched SPF-2 and non-SPF macaques that were housed under identical conditions revealed distinct differences in certain immune parameters suggesting that chronic pathogen exposure modulated host immune responses. All non-SPF macaques were infected with RhCMV, suggesting that chronic RhCMV infection was a major contributor to altered immune function in non-SPF macaques, although a causative relationship was not established and outside the scope of these studies. Further, we showed that immunological differences between SPF-2 and non-SPF macaques were already apparent in adolescent macaques, potentially predisposing RhCMV-infected animals to age-related pathologies.

**Conclusions:**

Our data validate rhesus macaques as a relevant animal model to study how chronic viral infections modulate host immunity and impact immunosenescence. Comparative studies in SPF-2 and non-SPF macaques could identify important mechanisms associated with inflammaging and thereby lead to new therapies promoting healthy aging in humans.

**Electronic supplementary material:**

The online version of this article (doi:10.1186/s12979-015-0030-3) contains supplementary material, which is available to authorized users.

## Background

Immune development and aging are dynamic processes that are governed by many distinct, yet interacting factors. Worldwide, as lifespans are generally increasing, there is a concomitant increase in age-related morbidities, particularly cardiovascular diseases, increased susceptibility to seasonal infections, cancers, and neurodegenerative disorders. A unifying link between the higher rates of these disparate diseases observed in aging populations is the progressive decline in immune functions. This phenomenon has been termed immunosenescence. The etiologies of immunosenescence are incompletely defined. A central driving force in the decline of immune function is the inherent process of progressive thymic involution, which results in an extremely restricted generation of naïve T cells by the fifth or sixth decades of life. However, studies over the past several years have emphasized the critical importance of extrinsic factors, which when layered over thymic involution, can further enhance immune dysfunction and thereby increase the rates of morbidity and mortality in aging individuals.

Several hypotheses have been presented to explain the mechanistic basis for increased disease susceptibility associated with aging. A main focus has been on the age-related development of a proinflammatory state, termed inflammaging. Although multiple studies have demonstrated increased production of inflammatory cytokines with age [1,2], the causes for inflammaging are likely multifactorial and remain to be determined. For example, polymorphisms in genes for IL-6, IL-10, and TLR4 have been associated with increased inflammation during aging [3-10]. This association was more pronounced in men than in women suggesting that hormones may influence the aging process. Indeed, age-related alterations of the endocrine system have been implied in altered immune responses of aging individuals (reviewed in [7]).

An alternative theory points to chronic infections as major driver in immunosenescence [11], and, in fact, the process of immunosenescence and inflammaging may be inextricably intertwined. Among the various known chronic viral pathogens, human cytomegalovirus (HCMV) in particular has been suggested to be associated with reduced immune function in older individuals [12-15]. HCMV is ubiquitous throughout the world with adult seroprevalence rates of 50-100%. HCMV, a member of the *Herpesvirales* order of viruses, is considered to be a virus with low pathogenic potential in immunocompetent hosts, but like all *Herpesvirales* members, it establishes lifelong persistence within the infected individual. Persistence is characterized by the presence of cells harboring latent HCMV genomes that periodically and asymptomatically reactivate to produce progeny virions that can be secreted in bodily fluids. The repeated and persistent antigenic stimulation of the immune host by HCMV, despite its low pathogenic potential, results in a unique virus-host relationship unlike that of any other virus, and this relationship has important clinical implications in relation to immunosenescence. A seminal study by Sylwester et al. quantified the CD4^+^ and CD8^+^ T cell responses to the HCMV proteome in 32 healthy long-term HCMV-infected individuals [16]. On average, almost 10% of memory CD4^+^ and CD8^+^ T cells are specific to HCMV antigens, far exceeding in magnitude the frequency of antigen-specific T cells to any other pathogen [17-20]. Other studies have indicated that there are progressive oligoclonal expansions of some of the HCMV-specific T cell populations. The majority of HCMV-specific effector memory cells are terminally differentiated and show reduced function, and are therefore considered immunosenescent [13]. At the same time, the persistence of antigens will continuously induce stress signals promoting inflammation [13]. Although seminal studies like the OCTA, NONA, and NHANES [21-24] cohort studies have clearly shown a statistical association between aging, reduced immune function, limited responsiveness to vaccines, increased C-reactive protein (CRP) levels, and HCMV seroprevalence, cause and effect cannot be conclusively distinguished [11,13,21,25-28]. An alternative hypothesis proposes that the vast expansion of HCMV-specific T cells consumes the available finite “niche” for immune cells and interferes with the *de novo* development of effector and memory cells targeting other pathogens or vaccines.

The underlying mechanisms of and associations between chronic infections and immune function could be best determined and tested in an animal model that closely recapitulates characteristics of human development, physiology, immunology, virology, and longevity. We propose that rhesus macaques represent such a model. Wild and captive populations of rhesus macaques are ubiquitously infected with rhesus cytomegalovirus (RhCMV), as well as other persistent pathogens [29]. Furthermore, RhCMV-infected monkeys show similar age-associated immune changes as those observed in HCMV-infected humans, including decreased CD4^+^ to CD8^+^ T cell ratios and the accumulation of terminally differentiated CD8^+^CD28^−^ T cells [30-32]. In captivity, these animals live for an average of 25 years and a maximum of approximately 40 years, whereas macaques in the wild generally live 12–18 years [30,33,34]. Macaques undergo similar age-related developmental, hormonal and immunological changes, but in an accelerated fashion compared to humans due to their shorter lifespan.

The current study aimed to demonstrate that the rhesus macaque model of HCMV persistence could be utilized as a relevant nonhuman model to study chronic infection-induced alterations of immune responsiveness as it applies to aging humans. Taking advantage of the unique resource of rhesus macaques that were bred and raised to be Specific Pathogen Free (SPF-2 macaques) for specific viruses (described below), we compared general age-associated changes in peripheral blood populations and in T cell function between SPF-2 and non-SPF macaques from birth until adulthood. We further investigated how immune responses to RhCMV evolve over the lifetime of a chronically infected animal in relation to virological parameters, and whether immune cell populations of RhCMV-infected animals would display distinct functional characteristics compared to age-matched SPF animals without RhCMV infection. Our results show that some age-related changes in major blood cell populations from birth to adulthood are common to both SPF-2 and non-SPF macaques. In addition though, our study clearly demonstrates that chronic RhCMV infection modulates immune development over the lifetime of the host. These altered responses were already evident in adolescent RhCMV-infected macaques, thereby potentially predisposing these non-SPF animals for inflammaging in late adulthood. These data support the idea that rhesus macaques could serve as a model system to study inflammaging, and provide a foundation for future studies to delineate specific mechanisms of RhCMV-induced immunosenescence with the long-term goal of developing therapeutic interventions, targeted to young adults, to prevent the onset of inflammaging.

## Results

### Immunological changes in peripheral blood cell population of SPF-2 macaques from birth to adulthood

An important first step towards our long-term goal of using the rhesus macaque model of RhCMV infection as a model system of health over the lifespan was to assess the influence of two comorbidities of immune function in humans: (i) maturation and aging of the immune system, and (ii) chronic exposure to persistent viral pathogens. Our approach was to characterize age-associated changes in basic immune cell populations and their functions in the presence or absence of infection with simian herpesviruses in general, and RhCMV in particular. There is ample evidence from human natural history studies that microbes that establish lifelong persistent infections in immune hosts, most notably HCMV, can profoundly alter leukocyte responses, which could potentially confound interpretation of changes in leucocyte populations associated with aging in rhesus macaques. Accordingly, we assessed age-related changes in major peripheral blood populations from birth to adulthood in SPF-2 macaques. We then tested whether non-SPF macaques underwent similar changes in immune populations with age, and whether altered immune parameters could be correlated to RhCMV infection status.

**Figure 1:**
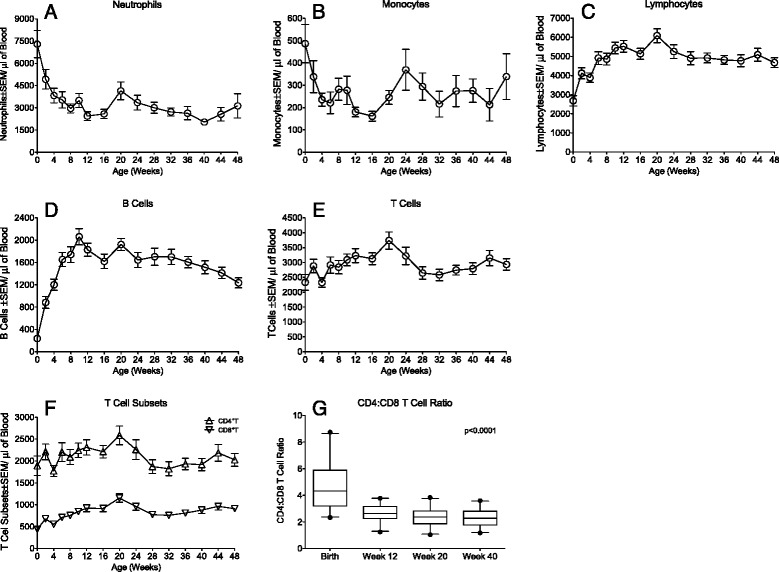
Longitudinal changes in major blood population during the first year of life. The graphs show longitudinal changes in the absolute frequencies of neutrophils **(A)**, monocytes **(B)**, or lymphocytes **(C)** in peripheral blood of SPF-2 infant macaques from birth to 1 year. Panels **(D)** to **(F)** illustrate the changes of B cells, T cells, and the CD4^+^ and CD8^+^T cell populations, respectively, in the first year of life. Data are represented as mean absolute numbers of 23 SPF-2 infants ± SEM per microliter of blood. Panel **(G)** shows the significant decline in the CD4:CD8 T cell ratio from birth to 3 months of age, after which the ratio remained stable throughout week 40. Boxplot horizontal bars indicate the median value in each age group.

Both acute and long-term changes were observed in the dynamics of leukocyte subsets during the rhesus macaque lifespan. During infancy (0–1 year), the numbers of neutrophils and monocytes appeared to decline. Indeed, 1-year old macaques had significantly lower neutrophil numbers than newborn macaques at birth (p < 0.0007, Figure 1A, Additional file 1: Table S1). The first 16 to 20 weeks of life represented the period of the greatest changes in cell numbers. During this period, mean neutrophil counts declined by 45%. During the same time period, monocyte counts declined by 49%, but in contrast to neutrophils, monocyte numbers rebounded and there was not a demonstrable difference between absolute monocyte counts at birth or 48 weeks (p = 0.1099; Figure 1B). Contemporaneous to these changes in neutrophil and monocyte counts, lymphocyte numbers increased (Figure 1C, Additional file 1: Table S1). The increase in lymphocytes was the result of both increasing B and T cell numbers (Figures 1D and E). Among the T lymphocytes, CD4^+^T cells represented the vast majority of T cells at birth, and, despite minor fluctuations, absolute CD4^+^T cell numbers did not significantly change throughout the first year (p = 0.2697; Figure 1F; Additional file 1: Table S1). However, because the CD8^+^T cell population expanded after birth (increase of mean CD8^+^T cells/μl blood from 441 at birth to 1150 at week 20; p < 0.0001) (Figure 1F, Additional file 1: Table S1), the percentage of CD4^+^T cells decreased concurrent with the rise in the CD8^+^T cell percentage (Additional file 1: Table S1). Therefore, the CD4:CD8 T cell ratio decreased from 4.8 at birth to 2.7 at week 12, stabilizing around 2.3 at week 20 and week 40 (Figure 1G). Thus, during the first few months of life, the various blood cell populations underwent obvious changes in their relative frequencies with cell numbers remaining more stable after 20 weeks of age (Figure 1). It is important to note that maintaining the animals in SPF-2 housing conditions absolutely restricted their potential exposure to multiple viruses normally endemic in the non-SPF animals. Previous seroepidemiological and molecular studies have demonstrated that infants born to non-SPF dams during the first year of life are increasingly likely to become infected with RhCMV, SFV, and RRV due to repeated exposure of bodily fluids containing infectious virus particles. Accordingly, changes in lymphoid subset profiles largely reflected age-related changes not influenced by these persistent viral pathogens (discussed in further detail below). In fact, nursery-reared infants born to non-SPF dams, and therefore experiencing minimal pathogen exposure, immunologically maturated identical to the SPF-infants (data not shown). The developmental changes occurred in both male and female infant macaques, and in both Indian and Chinese SPF-2 infants (data not shown), suggesting that macaque gender or genetic differences did not overtly affect early immune development of peripheral blood cells.

Age-related changes from infancy to adulthood in the same leucocyte populations were examined by cross-sectional analysis of SPF-2 rhesus macaques from different age groups (Table 1). Both neutrophils and monocytes were significantly higher (p < 0.0001 and p = 0.0354, respectively; see Table 1) in adolescent (3 to 5 years) compared to 1-year old infant macaques, whereas B and T lymphocytes (both CD4^+^ and CD8^+^ T cell subsets) were significantly lower (p < 0.0001). The CD4:CD8 ratio remained stable despite changes in CD4^+^ and CD8^+^ T cell numbers. The comparison of young adults (6 to 10 years) to adolescents (3 to 5 years) revealed higher monocyte numbers (p < 0.0001), but lower B (p = 0.0252) and T cell numbers (p = 0.0031). The reduction in CD3^+^T cells was due almost exclusively to a decline in CD4^+^T cells, resulting in an inversion of the CD4:CD8 ratio. The relative decline in CD4^+^ T cells in young adults compared to adolescents (Table 1) may have been a factor of sampling only few young adult SPF-2 animals available for this study. Nevertheless, the data demonstrate that there are continued significant changes in the frequencies of peripheral blood cell populations as animals age, changes that are also characteristic of many aging humans.

**Table 1:** **Peripheral blood mononuclear cell populations in SPF-2 rhesus macaques**

**Cell population**	**Median cell numbers/ μl blood (Minimum-Maximum)**				
	**Infants** ^**a**^	**Adolescents**	**Infants vs. Adolescents** ^**b**^	**Young Adults**	**Adolescents vs. Young Adults** ^**b**^
	**(n = 22)**	**(n = 30)**		**(n = 8)**	
Neutrophils	1952	10502	p < 0.0001	11274	NS^c^
	(990–4900)	(6667–18502)		(10032–19694)	
Monocytes	182	328	p = 0.0354	938	p < 0.0001
	(45–637)	(202–855)		(476–2030)	
Lymphocytes	4547	1949	p < 0.0001	710	p = 0.0014
	(2277–7314)	(1107–3088)		(342–2388)	
B Cells^d^	1464	474	p < 0.0001	224	p = 0.0252
	(649–2716)	(136–808)		(97–736)	
T Cells^e^	2759	1257	p < 0.0001	486	p = 0.0031
	(1373–4811)	(336–1899)		(194–1760)	
CD4^+^ T	1863	828	p < 0.0001	124	p < 0.0001
	(850–3598)	(459–1319)		(15–734)	
CD8^+^ T	820	397	p < 0.0001	363	NS
	(357–1843)	(209–1109)		(179–1026)	
CD4:CD8 Ratio	2.3	2.0	NS	0.4	p < 0.0001
	(1.2-3.6)	(0.7-3.8)		(0.1-0.8)	

^a^age: 40 weeks; ^b^Group comparison was performed by Mann–Whitney test; ^c^NS = not significant; ^d^B cells are defined as CD20^+^ lymphocytes; ^e^T cells are defined as CD3^+^ lymphocytes.

### Effect of pathogen exposure on age-related changes in immune cell populations

Having defined the phenotypic changes in peripheral blood T and B cell populations from infancy to young adulthood of SPF-2 animals, similar analyses were performed in adolescent and aged non-SPF macaques. Generally, analogous changes in peripheral blood lymphocyte populations were observed between non-SPF adolescent and adult macaques to those that were observed in SPF-2 animals (Table 2). With the exception of monocytes, there were significant decreases in almost all leucocyte populations, including neutrophils, B cells, total CD3^+^T cells, and within the CD4^+^ and CD8^+^ T cell subsets (Table 2). However, SPF-2 adolescent macaques had significantly (p < 0.0001) lower lymphocyte numbers than the non-SPF adolescent macaques (median: 1949/μl blood vs. 3037/μl blood, respectively; Table 3). Lower lymphocyte numbers were likely the result of lower median B cell numbers in the SPF-2 (474 /μl blood) compared to non-SPF (1445 /μl) adolescent macaques (p < 0.0001), because total median T cell numbers did not differ significantly between the groups (1257 vs. 1155 in SPF-2 and non-SPF adolescents, respectively; Table 3). Despite comparable T cell numbers, distinct from the SPF-2 macaques, significant reductions in both the CD4^+^T (p < 0.0001) and the CD8^+^T cell compartment (p = 0.0139) were observed in the non-SPF animals. This also resulted in a significant reduction of the CD4:CD8 T cell ratio (p < 0.0001), although the inversion noted in SPF-2 animals was not observed in non-SPF macaques (Tables 2 and 3). As defined below (Materials and Methods), the infectious distinction between SPF-2 and non-SPF populations was that SPF-2 animals were uninfected with RhCMV, herpes B virus, RRV, and SFV. Since both SPF-2 and non-SPF adolescent and adult study animals were housed outdoors under identical housing conditions, it is highly probable that both cohorts were exposed to the identical spectrum of microbes endemic in the environment of the CNPRC. Infant outdoor-housed non-SPF animals were not included in the comparative analysis because there is a differential median age of seroconversion to RhCMV, SFV, and RRV during the first year of life in outdoor-housed animals [35].

**Table 2:** **Peripheral blood populations in non-SPF macaques**

**Cell population**	**Median cell numbers/ μl blood (Minimum-Maximum)**		
	**Adolescents**	**Adults**	**Adolescents vs. Adults** ^**a**^
	**(n = 50)**	**(n = 100)**	
Neutrophils	9576	8280	0.0119
	(1936–21156)	(2520–20169)	
Monocytes	392	326	NS^b^
	(70–1620)	(42–1494)	
Lymphocytes	3037	1248	p < 0.0001
	(756–8073)	(262–3888)	
B Cells	1445	366	p < 0.0001
	(269–4317)	(69–1341)	
T Cells	1155	745	p < 0.0001
	(311–3730)	(103–2798)	
CD4^+^ T	753	433	p < 0.0001
	(195–2202)	(44–1606)	
CD8^+^ T	310	250	p = 0.0139
	(99–1401)	(43–1276)	
CD4:CD8 Ratio	2.4	1.8	p < 0.0001
	(1.4-4.0)	(0.2-4.2)	

^a^Data were compared by Mann–Whitney test. ^b^NS = non-significant.

**Table 3:** **Peripheral blood populations in adolescent SPF-2 and non-SPF macaques**

**Cell population**	**Median cell numbers/ μl blood (Minimum-Maximum)**		
	**SPF-2 Adolescents**	**Non-SPF Adolescents**	**SPF-2 vs. Non-SPF** ^**a**^
	**(n = 30)**	**(n = 50)**	
Neutrophils	10502	9576	NS^b^
	(6667–18502)	(1936–21156)	
Monocytes	328	392	NS
	(202–855)	(70–1620)	
Lymphocytes	1949	3037	p < 0.0001
	(1107–3088)	(756–8073)	
B Cells	474	1445	p < 0.0001
	(136–808)	(269–4317)	
T Cells	1257	1155	NS
	(336–1899)	(311–3730)	
CD4^+^ T	828	753	NS
	(459–1319)	(195–2202)	
CD8^+^ T	397	310	p = 0.0247
	(209–1109)	(99–1401)	
CD4:CD8 Ratio	2.0	2.4	p = 0.0132
	(0.7-3.8)	(1.4-4.0)	

^a^Data were compared by Mann–Whitney test. ^b^NS = non-significant.

### T cell and B cell memory development in SPF-2 versus non-SPF rhesus macaques

**Figure 2:**
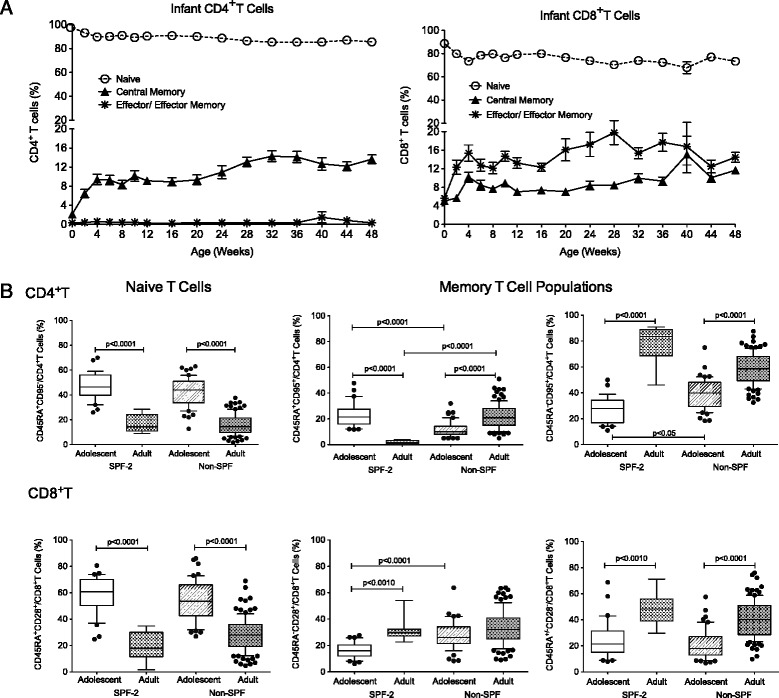
Memory T cell development*.* Panel **A**. Age-related changes in naïve and memory CD4^+^ and CD8^+^ T cell populations from birth to 1 year of age, expressed as mean percentages ± SEM, of 23 SPF-2 infants are shown. The markers CD28 and CD95 were used to define naïve (CD28^+^CD95^−^; open circles), central memory (CD28^+^CD95^+^; filled triangles), and effector/effector memory (CD28^−^CD95^+^; stars) T cell populations. Panel **B**. Naïve and memory T cell populations in SPF-2 and non-SPF adolescent (white and grey striped bars, respectively) and adult (white dotted and grey-dotted bars, respectively) macaques. The box-and-whisker plots show the 10–90 percentile range of the data of 30 SPF-2 adolescents, 8 SPF-2 young adults, 50 non-SPF adolescents and 100 adult non-SPF macaques. Significant differences between age groups were determined by one-way ANOVA and are indicated as p values.

The age-related changes in lymphocyte population frequencies were accompanied by immune maturation. As expected, the majority of CD4^+^ and CD8^+^T cells of SPF-2 animals expressed a naïve phenotype at birth (Figure 2A). Throughout the first year, naïve T cells (CD3^+^CD45RA^+^CCR7^+^ or CD3^+^CD28^+^CD95^−^) declined, while the percentage of both central memory (T_CM_) and effector/effector memory (T_E/EM)_) T cells increased. The development of memory CD8^+^T cells was more pronounced with close to 15% of CD8^+^T cells expressing a T_E/EM_ phenotype at 1 year compared to less than 1% of CD4^+^T cells (Figure 2A; Additional file 2: Table S2). Memory T cells further increased in adolescent animals and in young SPF-2 adults, as described for humans, memory T cells represented the majority of CD4^+^ and CD8^+^T cells (Figure 2B).

Similar to SPF-2 macaques, non-SPF adult macaques had fewer naïve and more memory CD4^+^ and CD8^+^T cells compared to adolescent macaques (Figure 2B). However, we noted some differences between SPF-2 and non-SPF macaques within the memory T cell subset distribution. Specifically, adolescent non-SPF macaques had higher frequencies of CD45RA^−^CD95^+^CD4^+^T cells and CD45RA^−^CD28^+^CD8^+^T cells (Figure 2B). Adult non-SPF macaques had significantly higher frequencies of CD45RA^+^CD95^+^CD4^+^T cells compared to SPF-2 macaques (mean percentages of 1.8% vs. 22%, respectively; p < 0.0001). Increased memory CD4^+^T cell populations in adolescent non-SPF compared to SPF-2 macaques were consistent with higher frequencies of CD4^+^T cells expressing CCR5, an activation marker that is absent on naïve T cells (Table 4). In contrast, CD25^+^CD4^+^T cells occurred at slightly higher percentages in SPF-2 adolescents. The latter could represent higher numbers of regulatory CD4^+^T cells in SPF-2 animals; however, because CD25 is also upregulated on activated T cells, and we did not assess functional parameters, this interpretation remains speculative. Higher frequencies of CD38^+^CD8^+^T cells in SPF-2 adolescents is likely related with the reduced memory CD8^+^T cell development in SPF-2 compared to non-SPF adolescent macaques (see Figure 2B), because CD38 is expressed on almost all infant T cells at birth and then declines slowly.

**Table 4:** **T cell activation**

**Marker**	**Mean cell numbers ± SEM / μl blood**				
	**SPF-2 Adolescents**	**Non-SPF Adolescents**		**Non-SPF Adults**	
			**SPF-2 vs. Non-SPF Adolescents** ^**a**^		**Non-SPF**
					**Adolescents vs. Adults** ^**a**^
**CD4** ^**+**^ **T**					
CD25	15.89 ± 0.66	13.58 ± 0.45	p = 0.0015	13.88 ± 0.50	NS
CCR5	33.77 ± 2.12	62.92 ± 5.26	p < 0.0001	46.60 ± 3.86	p < 0.0010
CXCR3	ND^b^	310.80 ± 20.80		221.30 ± 12.40	p < 0.0001
HLA-DR	15.48 ± 1.29	13.27 ± 1.60	NS^c^	7.55 ± 0.54	p < 0.0001
HLA-DR^+^CD25^+^	17.03 ± 5.49	5.00 ± 0.50	p = 0.0180	2.90 ± 0.27	p < 0.0001
**CD8** ^**+**^ **T**					
CD38	388.50 ± 25.54	233.60 ± 25.72	p < 0.0001	107.30 ± 10.39	p < 0.0001
HLA-DR	14.94 ± 1.40	12.55 ± 1.42	NS	9.33 ± 0.76	p = 0.0385
CD38^+^HLA-DR^+^	14.36 ± 1.34	9.12 ± 1.27	p < 0.0100	5.25 ± 0.66	p < 0.0010

^a^Data between groups were compared by Mann–Whitney analysis. ^b^ND = not determined; ^c^ NS = not significant.

**Figure 3:**
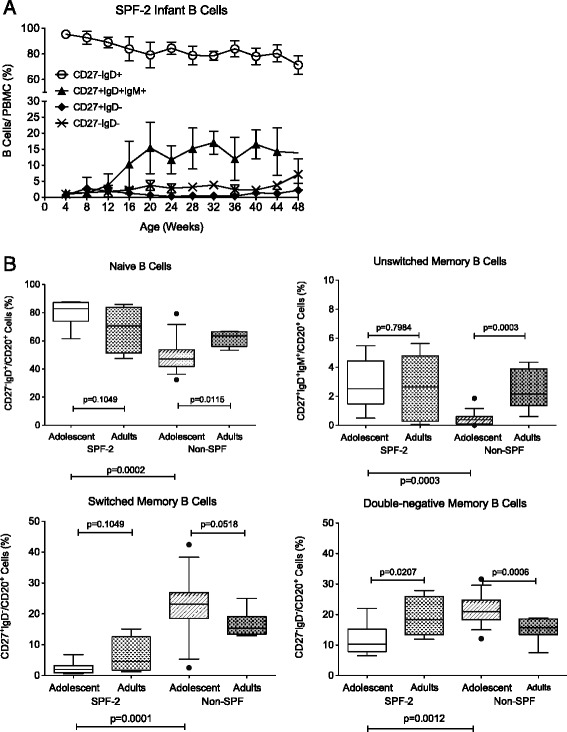
B cell differentiation in SPF-2 macaques with age*.* Panel **A**: The normal developmental changes in peripheral blood B cell populations from birth to 1 year of age in SPF-2 infant macaques (n = 16). Shown are mean values ± SEM for naïve, unswitched, isotype-switched and double-negative memory B cell populations based on phenotypic characterization using CD27 and IgD. Panel **B** illustrates age-related changes in B cell populations between infants at 40 weeks of age, adolescent (n = 8) and adult SPF-2 macaques (n = 8). The legend of the plots is as described in Figure 2. Significant differences between age groups were determined by exact Mann–Whitney test and are indicated as p values.

A temporal relationship was also observed between B cell maturation and age. In the first year of life, there was a continuous decline in naïve B cells (CD19^+^CD20^+^CD27^−^IgD^+^) accompanied by a simultaneous increase in unswitched (CD27^+^IgD^+^IgM^+^) and switched memory B cells (both CD27^+^IgD^−^ and double negative IgD^−^CD27^−^ B cells) (Figure 3A). As expected, frequencies of all memory B cell populations were higher in adolescent and adult macaques than in 1-year old infants independent of SPF-2 status (data not shown). Interestingly, adolescent non-SPF macaques had significantly higher frequencies of switched CD27^+^IgD^−^ (p = 0.0001) and double–negative CD27^−^IgD^−^ (p = 0. 0012) memory B cells, but fewer naïve B cells and unswitched memory B cells than SPF-2 adolescent macaques (p = 0.0002 and p = 0.003, respectively; Figure 3B). Although similar trends were observed for naive and switched memory B cell populations in young adult SPF-2 and adult non-SPF macaques, these differences did not reach statistical significance (p > 0.05); likely the result of the low animal numbers (n = 8) and the overall younger age of adult SPF-2 (6 to 10 years) compared to non-SPF adult macaques (12 to 20 years).

Thus, in addition to comparable age-related changes in B and T cell populations in SPF-2 and non-SPF macaques, there were distinct differences in T and B cell maturation phenotypes, suggesting that both age and pathogen exposure influenced T and B cell maturation.

### Differences in T cell function between SPF-2 and non-SPF macaques

**Figure 4:**
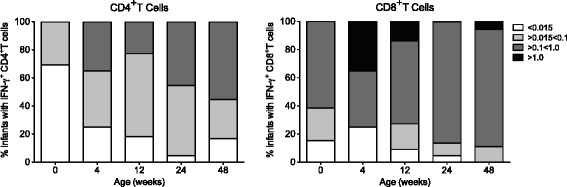
Age-dependent increase in infant IFN-γ CD4^+^T cell responses. The ability of CD4^+^ and CD8^+^T cells to produce IFN-γ in response to *in vitro* PMA/ionomycin stimulation was assessed in 23 infant SPF-2 macaques at various time points from birth to 48 weeks of age using flow cytometric analysis. The percentage of infant macaques with a positive IFN-γ response in CD4^+^ (left graph) or CD8^+^T cells (right graph) is shown for the time of birth (week 0), and for weeks 4, 12, 24, and 48 of age. Bars at a specific time point show the percentage of animals with a positive CD4^+^ or CD8^+^T cell response in less than 0.015% (white), between 0.015% and 0.1% (light grey), between 0.1% and 1.0% (dark grey), and in more than 1.0% (black) of the CD4^+^ or CD8^+^T cell population. Additional file 3: Table S3 contains the extended data set used for the graphical representation here. Note the age-dependent increase in the percentage of SPF-2 infants with higher frequencies of IFN-γ positive CD4^+^T cells. At birth, about 70% of infants have no or only a weakly positive IFN-γ response in CD4^+^ T cells (<0.015%), whereas at 48 weeks of age 50% of the infants show responses >0.1% (p = 0.002; Additional file 3: Table S3). In contrast, the magnitude of IFN-γ responses in CD8^+^T cells did not differ between newborn and 1 year old SPF-2 infant macaques (Additional file 3: Table S3).

To establish whether the phenotypic changes indicative of immune maturation with age were associated with altered T cell function, we measured non-specific T cell responses in the different age groups of SPF-2 animals. Consistent with the Th2-bias of infant CD4^+^T cell response [36,37], prior to 4 weeks of age infants had low frequencies of CD4^+^T cells producing IFΝ−γ, the principle Th1 characterizing cytokine, in response to polyclonal T cell stimulation (Figure 4, Additional file 3: Table S3). The percentage of IFN-γ-producing CD4^+^T cells increased from <0.015% in the first month to >0.1% of total CD4^+^T cells by 40–48 weeks of age (Additional file 3: Table S3; p = 0.002). In contrast, CD8^+^T cell IFN-γ responses were consistently detected throughout the first year and were of comparable magnitude ranging from 0.1 to 1% of all CD8^+^ T cells (Figure 4, Additional file 3: Table S3, p = 0.111), implying that CD4^+^T cell-intrinsic factors may interfere with IFN-γ production in neonates.

**Figure 5:**
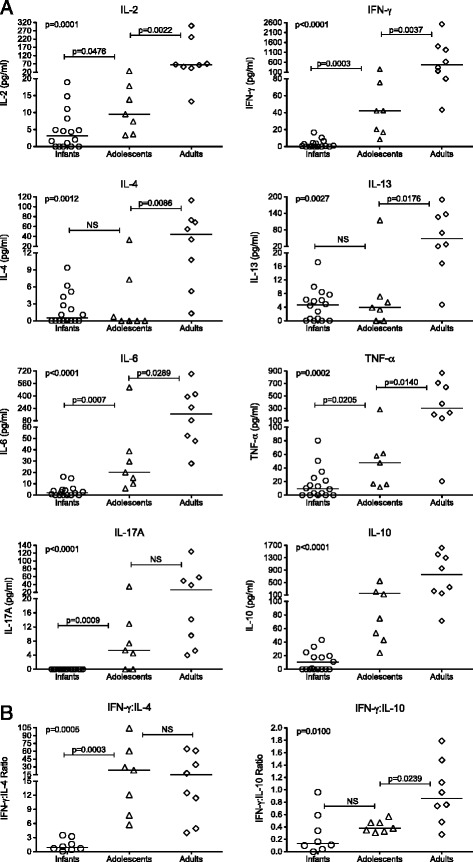
TCR-induced cytokine responses in PBMC of SPF-2 macaques of different age groups. Panel **(A)**: The various graphs depict specific cytokines produced by PBMC of infant (n = 16), adolescent (n = 7) and young adult SPF-2 macaques (n = 8) after 24 hours of stimulation with anti-CD3 and anti-CD28 antibodies. Data are expressed in pg per milliliter (pg/mL) of culture supernatant. Panel **(B) **shows age-related changes in the ratios of IFN-:IL-4 and IFN-γ:IL-10. Cytokine levels between the three distinct age groups were compared by one-way ANOVA and statistically significant differences are indicated by p values. Each symbol represents an individual animal; horizontal lines indicate the median of the age-group.

To measure more physiologically relevant responses, we stimulated SPF-2 infant PBMC with anti-CD3 and anti-CD28 antibodies to activate T cells directly through the T cell receptor (TCR). Supernatants were collected at 24 hours and analyzed for cytokines important in T cell activation and CD4^+^T helper cell lineage differentiation. This experiment included a group of 3 to 9 week old SPF-2 infants (n = 8) and a second group of 10 to 16 week-old infant macaques (n = 8). As we did not detect statistically significant differences in cytokine responses between these two groups (data not shown), we combined the data from both infant groups and compared them to cytokine levels induced by TCR stimulation in SPF-2 adolescent (n = 7) and SPF-2 young adult macaques (n = 8). There was an age-dependent increase in several cytokine responses to TCR stimulation from infant to adult macaques (Figure 5A). In addition to these quantitative changes, the quality of the response differed between age groups. Consistent with the very low IFN-γ response of infants, the ratio of IFN-γ:IL-4 was lower in infant compared to adolescent and young adult SPF-2 macaques (Figure 5B). Similarly, and despite increasing production of both IL-10 and IFN-γ with age, the IFN-γ:IL-10 ratio was also significantly lower in infants.

**Figure 6:**
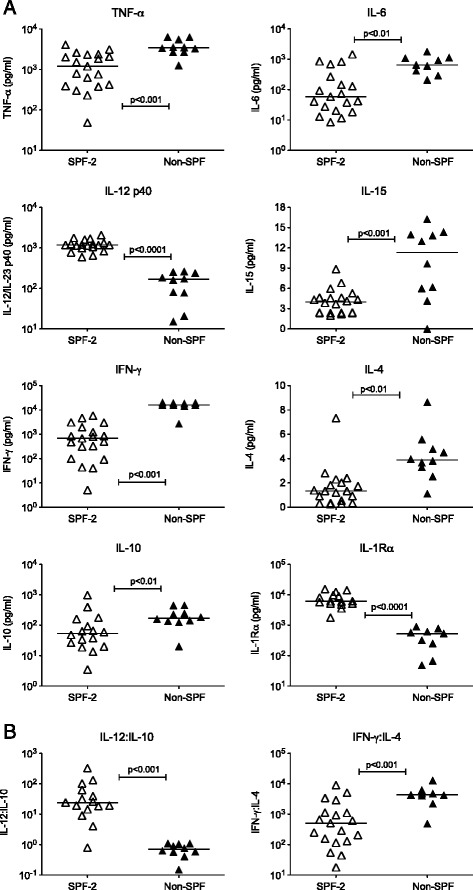
Cytokine responses of adolescent SPF-2 and non-SPF macaques to ConA stimulation. Panel **(A)**. The individual graphs show the production of specific cytokines in PBMC of SPF-2 (open triangles; n = 20) or non-SPF adolescent macaques (filled triangles; n = 10) after in vitro stimulation with Con A. Cytokines were measured in culture supernatants at 48 hours. Capped lines with p values indicate statistically significant differences between the two groups determined by Mann–Whitney test. Each symbol represents an individual animal; horizontal lines indicate the median of the age group. Panel **(B)** shows how the cytokine ratios of IL-12:IL-10 and IFN-γ:IL-4 differ between adolescent SPF-2 and non-SPF macaques.

To validate our earlier conclusion that, in addition to age, pathogen exposure influences T cell maturation and function, we compared cytokine response to ConA stimulation between SPF-2 and non-SPF adolescent macaques. Most proinflammatory cytokines, including TNF-α, IL-6 (Figure 6A), IL-1β, and TNF-β (data not shown) were expressed at higher levels in non-SPF than in SPF-2 adolescent macaques. Although SPF-2 adolescents produced significantly more IL-12p40 and had a higher IL-12:IL-10 ratio than non-SPF adolescents (Figure 6B), the lower IFN-γ response of SPF-2 adolescents (Figure 5A) resulted in a significantly lower ratio of IFN-γ: IL-4 compared to non-SPF adolescent macaques (Figure 6B). Thus, the overall cytokine response in non-SPF adolescents was indicative of an immune-activating, inflammatory bias compared to SPF-2 adolescent macaques, and this apparent bias was maintained into adulthood. Although anti-inflammatory cytokines (e.g. IL-10, IL-1Rα) and cytokines associated with Th2 responses (IL-4, IL-9) were also produced at higher levels in adult compared to adolescent non-SPF animals, inflammatory cytokines such as TNF-α and the Th1-associated chemokine RANTES were increased concurrently (Table 5).

**Table 5:** **Cytokine responses to** ***in vitro*** **ConA stimulation**

**Cytokine**	**Median cytokine levels in pg/ ml (Minimum-Maximum**)				
	**SPF-2 Adolescents**	**Non-SPF Adolescents**	**SPF-2 vs. Non-SPF Adolescents** ^**a**^	**Non-SPF Adults**	**Non-SPF Adolescents vs. Adults** ^**a**^
***Inflammatory***					
IL-6	58.0	647.7	p < 0.0500	1267.0	NS^b^
	(8.6-1451)	(207.6-1778)		(268.2-2253)	
TNF-	1198.0	3436.0	p < 0.0010	6181.0	p < 0.0500
	(48.2-4103)	(1260.0-6368.0)		(2246–9517)	
IL-1β	23.4	113.7	p < 0.0010	226.3	NS
	(0.3-71.4)	(25.5-259.0)		(80.9-795.9)	
TNF-β	334.7	1185.0	p < 0.0010	753.2	NS
	(31.5-1089.0)	(104.6-2307.0)		(232.3-1465.0)	
IL-12p40	1174.0	167.3	p < 0.0001	215.8	NS
	(592.9-2068.0)	(15.1-260.8)		(96.2-606.6)	
IL-15	4.0	11.3	p < 0.001	11.1	NS
	(1.9-8.9)	(0–16.2)		(7.6-15.6)	
CXCL-10	33.0	13.1	p < 0.0001	10.6	NS
	(16.2-45.0)	(3.0-23.4)		(0.1-22.0)	
***Anti-Inflammatory***					
IL-10	53.5	171.2	p = 0.0251	489.7	p = 0.0115
	(3.5-974.9)	(20.0-453.0)		(72.3-978.1)	
IL-1Rα	6073.0	529.7	p < 0.0001	937.8	p = 0.0279
	(1775–15352)	(48.2-887.4)		(266.6-3574.0)	
***Th differentiation***					
IL-2	2451.0	4892.0	NS	3511.0	NS
	(19.4-21229.0)	(40.6-7656.0)		(814.1-23975)	
IFN-γ	681.0	16058.0	p < 0.0010	19656	NS
	(5.2-5832.0)	(2704–20164)		(14315–20941)	
IL-4	1.3	3.9	p < 0.0500	9.3	p = 0.0015
	(0.3-7.3)	(1.1-8.6)		(4.2-21.7)	
IL-9	259.2	20.5	p < 0.0001	38.2	p = 0.0089
	(126.2-512.0)	(9.8-47.9)		(24.2-103.3)	
***Chemokines***					
RANTES	11017.0	970.5	p < 0.0001	1818.0	p = 0.0089
	(800.3-37070)	(151.8-2041.0)		(930.2-3434.0)	
Eotaxin	13.3	21.3	p = 0.0301	21.7	NS
	(4.1-24.1)	(8.0-31.2)		(1.5-25.5)	
***Cytokine Ratios***					
IL-12:IL-10	24.1	0.7	p < 0.0010	0.3	NS
	(0.8-326)	(0.2-1.1)		(0.2-0.8)	
IFN-γ:IL-4	508.2	4329.0	p < 0.0010	2032.0	p = 0.0350
	(17.8-8997.0)	(484.6-12917)		(886.0-4871.0)	
IL-1β:IL-1Rα	0.004	0.36	p < 0.0001	0.27	NS
	(0.0-0.0096)	(0.04-3.69)		(0.06-0.88)	
RANTES:Eotaxin	611.3	41.0	p < 0.0001	79.8	p = 0.0435
	(64.1-8074.0)	(16.7-158.1)		(52.9-1353.0)	

^a^Data were compared by Mann–Whitney test. ^b^NS = non-significant.

### RhCMV immunity in adolescent and adult non-SPF macaques

RhCMV is one of the most common pathogens present in non-SPF rhesus macaque colonies. Thus, we wanted to determine whether RhCMV infection status was associated with any of our observed changes in immune development and maturation between SPF-2 and non-SPF macaques.

**Figure 7:**
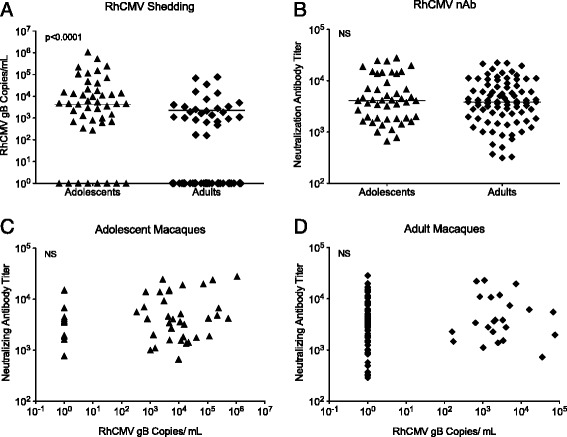
Viral and immune parameters of RhCMV infection*.* Panel **A** shows the quantity of RhCMV shed orally by adolescent (filled triangles; n = 50) or adult (filled diamonds; n = 100) non-SPF macaques. Each symbol represents an individual animal; horizontal lines indicate the median of the age group. In Panel **B**, neutralizing plasma antibody titers for RhCMV-infected adolescent and adult macaques are shown. The legend is similar to Panel **A**. Neutralizing antibodies were correlated with oral RhCMV shedding in adolescent (Panel **C**) or adult (Panel **D**) macaques. Statistically significant differences are indicated by p values.

We previously documented that about 90% of conventional non-SPF macaques housed in outdoor corrals become infected with RhCMV between 5–12 months of age [38-40]. In agreement with these findings, in the current study, RhCMV shedding was detected in the saliva of most adolescent non-SPF macaques (Figure 7A). RhCMV shedding was more frequent (78% vs. 28%), and occurred at significantly higher titers (mean: 5.47 × 10^4^ copies/ml with 95% CI of mean from 6665 to 102833) in adolescent compared to adult non-SPF macaques (mean: 2.31 × 10^3^ copies/ml with 95% CI of mean from 348 to 4265) (Figure 7A). To confirm RhCMV infection in both shedding and non-shedding macaques, we performed serological testing. Adolescent and adult non-SPF macaques showed similar RhCMV gB–specific neutralizing antibody titers (Figure 7B), suggesting that once neutralizing antibody titers were induced, they persisted at moderate to high levels. This result, similar to HCMV in humans, is consistent with the lifelong persistence of RhCMV in the animals without causing disease, despite occasional virus shedding [38]. In the adolescent macaques that shed RhCMV at higher titers and more frequently, neutralizing antibody titers were weakly associated with RhCMV shedding using Pearson correlation, but not when a non-parametric Spearman’s rho test is applied (Figure 7C). An association was not present in adult macaques who shed RhCMV at low titers and infrequently (Figure 7D).

**Figure 8:**
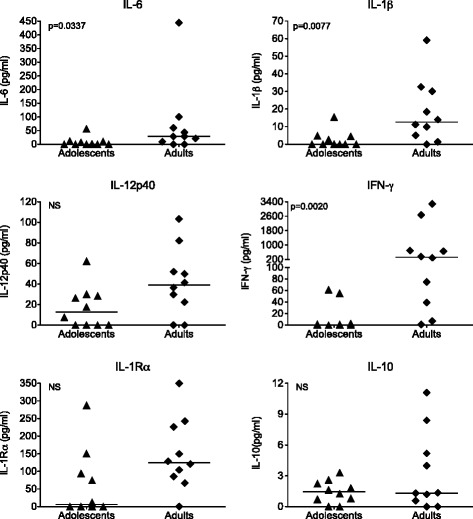
Cytokine responses to in vitro RhCMV stimulation*.* Each graphs represents the amount (pg/ mL) of a specific cytokine produced in response to RhCMV lysate after 48 hours of stimulation. Each symbol is representative of a single animal and horizontal bars indicate median cytokine levels for each age group. A total of 10 adolescent and 10 adult non-SPF macaques were included in the analysis. Statistically significant differences are indicated by p values.

In contrast to neutralizing antibody responses, RhCMV-specific T cell responses further increased with age. Most of the adolescent non-SPF macaques produced only a limited number of cytokines and only at low levels in response to in vitro RhCMV lysate stimulation (Figure 8). Consistent with the expansion of memory T cells in adult compared to adolescent non-SPF macaques, all adult RhCMV-infected macaques secreted multiple cytokines and at higher levels than adolescent RhCMV-infected macaques, although statistically significant age-related differences were only observed for IL-6, IL-1β, and IFN-γ (Figure 8). Median values of TNF-α and RANTES were also higher in adult than in adolescent macaques, but this difference did not reach statistical significance (data not shown). Thus, reminiscent of the more inflammatory response to the non-specific ConA stimulation in non-SPF compared to SPF-2 macaques, antigen-specific responses to RhCMV lysate in chronic RhCMV–infected macaques showed a proinflammatory cytokine bias.

## Discussion

The goal of the study was to validate the rhesus macaque model of RhCMV infection as a relevant animal model to study immune modulation of aging and immunosenescence by chronic viral infections. Towards this goal, we confirmed that rhesus macaques undergo similar changes in frequencies, maturation, activation, and function of peripheral blood cell populations from birth to adulthood as humans. The comparison of immune parameters in different age groups of SPF-2 and non-SPF macaques revealed that chronic pathogen exposure modulates host immunity.

In a first step to define normal developmental changes in immune cell populations and their function with age, we followed a cohort of SPF-2 infants from birth to 1 year of age. Our results show that the infant immune system undergoes tremendous changes after birth, with the most dramatic changes in cell frequencies occurring in the first 3–4 months of life. Memory T and B cell populations steadily increased throughout the first year, and these phenotypic changes were associated with functional changes. For example, the magnitude of IFN-γ responses in CD4^+^T cells increased in the first year of life. In contrast, the magnitude of CD8^+^T cell IFN-γ responses was comparable at birth and 1 year of age, and generally exceeded that of infant CD4^+^T cells, suggesting that factors intrinsic to CD4^+^T cells regulate IFN-γ production in CD4^+^ compared to CD8^+^ infant T cell populations. Interestingly, it has been previously reported that the IFN-γ promoter is hypermethylated in human infant CD4^+^T cells [41], and therefore future studies should determine whether epigenetic changes during the first year of life could explain the observed increase in IFN-γ production [42]. We acknowledge though that immune development is likely influenced by various factors. In infants specifically, rearing conditions can dramatically affect immune development and function. A recent study demonstrated that dam-reared, and therefore breast-fed infants, versus nursery-reared infant rhesus macaques show dramatic differences in the development of Th17 cells as a result of their distinct microbiota [43]. Thus, immune development and function in the early period of life from birth to 1 year might be uniquely regulated by a multitude of intrinsic and extrinsic factors. Nonetheless, our normative data on the frequencies of peripheral blood cell populations, and on T and B cell maturation in infant SPF-2 rhesus macaques are remarkably similar and consistent with normal developmental changes observed in human infants [44,45].

Considering the potential impact of infant rearing on immune development, and knowing that there is a differential median age of seroconversion to RhCMV, SFV, and RRV during the first year of life in outdoor-housed rhesus macaques, we evaluated the effect of chronic pathogen exposure on host immunity by comparing adolescent SPF-2 and non-SPF macaques. Both of these animal cohorts were housed outdoor under identical conditions and therefore exposed to identical microbes in the environment; thus, the major apparent distinction between SPF-2 and non-SPF adolescent macaques was their infection with RhCMV, SFV and RRV. In addition, we established whether pathogen-induced immune modulation in adolescent non-SPF macaques persisted into adulthood or whether aging resulted in additional immunological changes.

Our cross-sectional analysis of infant, adolescent and adult macaques suggests that both SPF-2 and non-SPF macaques experience a significant decrease in lymphocyte numbers with aging. Concurrently, the composition of the T and B cell populations changed. With increasing age, and presumably pathogen exposure, effector and memory T and B cells developed and T cell maturation was associated with functionally improved T cell responses. These age-related immunological changes were common to SPF-2 and non-SPF macaques, but differences in the quality and magnitude of these changes suggested that chronic pathogen exposure could modulate host immune responses. Specifically, memory T cells, in particular CD4^+^ memory T cells, occurred at higher levels in non-SPF than in SPF-2 adolescent and adult macaques. Similarly, adolescent and adult non-SPF animals had higher frequencies of isotype-switched memory B cells than SPF-2 macaques of the same age. These differences in specific lymphocyte subsets likely contributed to the observed distinct cytokine responses after mitogenic T cell stimulation. Non-SPF adolescent macaques produced higher levels of cytokines, in particular proinflammatory cytokines, compared to their age-matched SPF-2 adolescent macaques. It is plausible that the active replication of RhCMV in adolescent macaques, that was reflected in their more frequent shedding patterns of RhCMV in oral fluids, contributed to the more inflammatory cytokine profile of non-SPF compared to SPF-2 adolescent macaques. Moreover, these qualitatively distinct responses in adolescent macaques were indicative of early predisposition of adolescent RhCMV-infected animals to “inflammaging” in adult macaques, and supported our hypothesis that chronic RhCMV infection could broadly modulate host immune responses.

To corroborate this conclusion further and to assess the biological relevance of these findings, we evaluated how RhCMV infection status and associated virologic and immunologic markers related to the age-related changes specific for non-SPF macaques. More than 90% of conventionally housed rhesus macaques are infected with RhCMV by 1 year of age [38-40], and, similar to humans, RhCMV persists in rhesus macaques throughout their lifespan [31,38,40,46,47]. Indeed, all of the adolescent and adult non-SPF macaques in the current study tested seropositive for RhCMV. Interestingly, adolescent and adult non-SPF macaques had comparable titers of RhCMV-specific neutralizing plasma antibody titers, suggesting that, once elicited, neutralizing antibodies to RhCMV persist at relatively constant levels. Further, we concluded that the magnitude of the neutralizing antibody response was not dependent on antigenic load because we observed significantly higher and more frequent viral shedding in adolescent compared to adult RhCMV-infected macaques. Although, our studies were not designed to determine a causal relationship, we would speculate that the induction and development of RhCMV-specific antibodies in adolescent non-SPF macaques was a contributing factor in the development of more mature B cells in non-SPF compared to SPF-2 macaques. Memory T cell expansion is a well-described phenomenon in human HCMV infection and in RhCMV infection in rhesus macaques [31,48,49]. While we did not measure actual frequencies of RhCMV-specific memory T cells, we noted significantly higher effector CD4^+^T cell and increased CD28-negative CD8^+^T cell populations in non-SPF compared to SPF macaques. Consistent with memory T cell inflation, adult compared to juvenile non-SPF macaques produced higher cytokine levels in response to RhCMV stimulation.

To conclusively define the underlying mechanisms leading to inflammaging, future longitudinal and comparative studies between animals of SPF-2 and non-SPF colonies are needed. Such studies could address (i) how normal aging or immunosenescence differ from inflammaging, (ii) which processes regulate and control inflammaging, (iii) whether genetic factors contribute to inflammaging in chronically infected humans, (iv) whether RhCMV or other chronic infections induce epigenetic changes in immune cell populations, and (v) whether pathogen-induced immune modulation would be reversible if recognized earlier in life.

## Conclusions

The results of our study validate rhesus macaques as a relevant animal model to study how chronic viral infections modulate host immunity and impact immunosenescence. Immunological aging is reflected in similar phenotypic and functional changes of peripheral blood cell population in in SPF-2 and non-SPF macaques from birth throughout adulthood. The changes in the immune repertoire of SPF-2 and non-SPF macaques that were associated with aging and infectious burden of persistent viruses recapitulate changes in lymphoid subsets observed in aging and HCMV-infected humans.

The identification of noticeable differences between adolescent SPF-2 and non-SPF macaques suggests that chronic infections, in particular RhCMV infection, modulate host immune development and aging. The changes in lymphoid subsets are hallmarks of two non-mutually exclusive interpretations of virus-host interactions. (1) As the viruses that define SPF-2 status establish lifelong persistent infections, the observed changes between age-matched SPF-2 and non-SPF animals must be considered as a phenotype of viral persistence in an immune-competent host. (2) Both primary and persistent infections of all of the SPF-2 viruses are subclinical. Therefore, the distinctions in the lymphoid repertoire must be considered as a phenotype of protective immune responses.

The distinctions in the relative skewing towards Th2 versus Th1-type of responses in SPF-2 and non-SPF animals, respectively, are consistent with a viral type of adjuvant effect that enables the seeming conundrum in which the SPF-2 pathogens stimulate antiviral immune responses in immune competent hosts that protect against viral sequelae, yet fail to clear reservoirs of persistent virus.

Comparative studies in SPF-2 and non-SPF macaques could identify important mechanisms associated with inflammaging and thereby lead to new therapies promoting healthy aging in humans.

## Methods

### Animals

The rhesus macaques (*Macaca mulatta*) used in this study were part of the California National Primate Research Center (CNPRC) breeding colony on the University of California, Davis campus. The Institutional Animal Care and Use Committee of the University of California, Davis (UC Davis), which is fully accredited by the Association for Assessment and Accreditation of Laboratory Animal Care, approved all animal protocols in advance of any procedures. Rhesus macaques generally reach puberty around four years of age. For this study, cohorts of animals 3 to 5 years of age (adolescent to young adult) were evaluated and are referred in the text as adolescent macaques. Rhesus macaques can live up to 30 years at the CNPRC, and aged macaques are defined as ≥14 years of age.

Genetically outbred infant rhesus macaques (*Macaca mulatta*) of Indian (n = 13; 8 male and 5 female infants) or Chinese (n = 10; 8 female and 2 male infants) origin were obtained from the Specific Pathogen-Free Level 2 (SPF-2) colony at the California National Primate Center (CNPRC). SPF-2 status was defined as being seronegative for the SPF Level 1 viruses simian type D retrovirus (SRV), simian immunodeficiency virus (SIV), simian T cell lymphotropic/leukemia virus (STLV), and Cercopithecine herpesvirus 1 (CHV-1; herpes B virus), and in addition being uninfected with RhCMV, simian foamy virus (SFV), and rhesus rhadinovirus (RRV) [50]. SPF-2 infant macaques were reared indoors in the SPF-2 nursery. At the age of 9 months to 1 year, they were relocated to outdoor SPF-2 animal corrals and were not co-housed with non-SPF animals. SPF-2 adolescent macaques (n = 20; 3 to 4 years), and non-SPF adolescent (n = 50; 3 to 5 years) or adult non-SPF macaques (n = 100; 12 to 20 years) were of Indian origin and housed outdoors in half-acre corrals. In addition, a smaller cohort of 8 young adult SPF-2 macaques (6 to 10 years) was included, the maximum number and age of adult SPF-2 animals available at the time of the study. Representative of the CNPRC colony’s effort to increase breeding age of female macaques, the gender distribution was shifted toward female macaques that represented 65% of SPF-2 adolescent, 42% of non-SPF adolescent, and 70.5% of adult non-SPF macaques.

### Sample collection and processing

Animals were immobilized with (10 mg/kg) ketamine-HCl intramuscularly (IM) prior to all sample collections. Complete blood counts (CBC) were performed at each blood collection. Infant EDTA blood samples were collected longitudinally starting at birth and subsequently every month for one year. Plasma was removed from whole blood by centrifugation and stored at −80°C for antibody measurement. Peripheral blood mononuclear cells (PBMC) were isolated from whole blood via density centrifugation using Lymphocyte Separation Medium (LSM) (MP Biomedicals, Solon, OH) or Accu-Paque Lymphocyte separation media (Accurate Chemical & Scientific Corp., Westbury, NY) as described [51-53]. Due to the low blood volume that could be collected from infant macaques, and therefore relatively low cell counts, infant PBMC were used immediately for functional analyses. PBMC from adolescent and adult macaques were cryopreserved for functional assays and batch-analysis.

### Complete blood count (CBC)

EDTA blood samples were analyzed on a HORIBA Pentra 60+ electronic cell counter (HORIBA Diagnostics, Irvine, CA). A standard blood smear was prepared from the EDTA sample and stained with a Wright-Giemsa stain (Harleco, EM Scientific, Gibbstown, NJ). Manual 100-cell differentials were performed for the differentiation of white blood cells. The relative percentage of each cell type was obtained and then multiplied by the total white blood cell count to get the absolute numbers of each cell population.

### T cell receptor (TCR) stimulation of SPF-2 macaques

Microtiter tissue culture plates were coated overnight with 5 μg/ml of pure anti-CD3 (clone SP34-2) and 5 μg/ml pure CD28 (clone L293) in 100 μl of PBS/ well. The next day, plates were washed 3× with PBS. PBMC (1×10^6^ cells/ ml) were then plated at 100 μl/ well and cultured for 24 hours at 37°C and 5% CO_2_. Supernatants were collected and stored at −80°C until analysis using the Milliplex MAP Nonhuman Primate Cytokine Panel (EMD Millipore, Billerica, MA).

### Concanavalin A (ConA) and RhCMV antigen stimulation

PBMC (1.25 × 10^6^ /ml) from adolescent and adult macaques were resuspended in RPMI/10% FBS and stimulated with 25 μg/ml concanavalin A (Con A) or 25 μg/ml RhCMV lysate prepared from an infected cell extract exhibiting 100% cytopathic effect. Negative control cultures consisted of cells in media only. Cell culture supernatants were collected after 48 hours and were immediately analyzed in duplicates for multiple soluble markers using multiplex bead arrays (Bioplex Suspension Array System with Bioplex Manager 4.0 software by Bio-Rad Laboratories, Hercules, CA, or Milliplex MAP Nonhuman Primate Cytokine Panel by EMD Millipore).

### Flow cytometric analysis

Infant PBMC T cell or B cell populations were analyzed for activation or memory cell differentiation using standard surface staining protocols [51-53]. The ability of the CD4^+^ and CD8^+^ T cells to produce cytokines in response to PMA/ionomycin stimulation was evaluated using the following logical gating strategy: PBMC gate (FSC-A vs. SSC-A), singlets (FSC-H vs. FSC-A), T cells (CD3^**+**^CD4^**+**^ or CD3^**+**^CD8^+^), followed by gating for IFN-γ, IL-2 and/or TNF-α within the CD4^+^ or CD8^+^T cell populations. T cell activation and maturation were determined by including Ki67, HLA-DR, CD38, CCR5, CXCR3, CD45RA, CD28, and/or CD95 in the analysis. Samples were acquired on a FACS Aria flow cytometer (BD Biosciences, San Jose, CA).

In addition, infant PBMC were resuspended with RPMI 1640 media (Cellgro, Manassas, VA) supplemented with 10% heat-inactivated FBS (Cellgro, Manassas, VA) and L-glutamine-penicillin-streptomycin antibiotic cocktail (Sigma, St Louis MO) at 1.0 ×10^6^ cells/ml. Negative controls were cultured in media only. To evaluate polyclonal T cell responses, PBMC were stimulated with phorbol myristate acetate (PMA) at 10 ng/ml and ionomycin at 125 ng/ml (Sigma). The cells were incubated for a total of 12 hours at 37°C and 5% CO_2_ with Brefeldin A (3.0 μg/ml) being added after the first hour (eBioscience, San Diego, CA). At the end of the culture period, cells were stained for surface antibodies, fixed, and permeabilized for intracellular cytokine staining (see below) and as described previously [51-53]. A minimum of 300,000 events were acquired for T cell stimulation responses and 30,000 events for phenotyping studies. Data were analyzed using FlowJo Software (Tree Star, Ashland, Oregon) and Boolean gating strategies were applied when appropriate. Cytokine responses in CD4^+^ or CD8^+^ T cells were considered positive if their frequencies were at least 2 times higher than in unstimulated (media) PBMC and if their actual value was ≥0.01%. Data are reported as percentage of CD4^+^ or CD8^+^ T cells.

To analyze T cell maturation and activation in adolescent and adult macaques, whole blood samples were stained by direct labeling with the relevant antibody cocktails and processed using the Coulter TQ Prep System (Coulter Corporation, Hialeah, FL) to lyse red cells and fix the stained PBMC. Samples were acquired on a FACS Calibur and analyzed with CellQuest software (BD Biosciences, San Jose, CA).

### RhCMV antibody measurement

RhCMV neutralizing antibodies (nAb) were determined as described previously [51,52]. Data are reported as the reciprocal of the dilution that resulted in 50% neutralization (NT50).

### RhCMV quantitation

Oral saliva swabs were collected from both RhCMV-seropositive non-SPF adolescents and adults. RhCMV was quantitated as previously described [54,55]. Real-time PCR for RhCMV gB was performed using the following primer and probe pair: forward primer: 5′-TGC GTA CTA TGG AAG AGA CAA TGC-3′, reverse primer: 5′-ACA TCT GGC CGT TCA AAA AAA C-3′ (Invitrogen, Carlsbad, CA), and probe (5′-3′) TET-CCA GAA GTT GCG CAT CCG CTT GT-TAMRA (Applied Biosystems, Carlsbad, CA). RhCMV was quantitated based on a RhCMV gB plasmid standard curve spanning 10° to 10^6^ copies; the limit of detection being 10 copies. The viral load was calculated as RhCMV gB copies per ml for saliva.

### Statistical analysis

The data are presented as median values if not indicated otherwise. The Wilcoxon signed ranked test was applied to determine whether changes in absolute cell numbers or percentages of cell populations between birth and 1 year of age reached statistical significance. Results between two or more independent groups were compared by Student’s t-test or one-way ANOVA, respectively, using GraphPad Prism, version 5 (GraphPad, Inc., La Jolla, CA). Nonparametric Mann–Whitney tests or Kruskal-Wallis tests with Dunn’s multiple comparison adjustments were applied when we observed large variation within a group, which is not untypical due to the outbred nature of rhesus macaques. To test whether there was an age-dependent increase in IFN-γ producing CD4^+^ and CD8^+^T cells from birth to 1 year of age, not necessarily on an individual basis, but on a population basis, an exact statistical test was applied. For each animal and cytokine outcome, an early (week 0–4) and late (week 40–48) measurement was derived from the arithmetic mean of three scheduled measurements during the respective timeframes. These timeframes were chosen prior to conducting statistical analysis. An exact Wilcoxon signed-rank test was used to test the null hypothesis that the late - early differences have a median of 0. A Hodges-Lehmann estimate of location shift and corresponding 95% confidence interval were also computed using R version 2.15.1 (http://www.r-project.org). Statistical inference was performed for IFN-γ in CD4^+^ and CD8^+^T cells without adjustments for multiple testing.

## Additional files

Additional file 1:
**Peripheral blood cell populations in SPF infant rhesus macaques.**
Additional file 2:
**Memory T cell development in infant SPF-2 macaques.**
Additional file 3:
**IFN-γ responses in infant T cells.**
