# Supplementary material for: The interplay between immune maturation, age, chronic viral infection and environment
Source: Immun Ageing. 2015 May 9;12:3. doi: 10.1186/s12979-015-0030-3 (PMC4436863; doi:10.1186/s12979-015-0030-3)
Supplement: Additional file 1: — Peripheral blood cell populations in SPF infant rhesus macaques. [file 12979_2015_30_MOESM1_ESM.pdf]

Table S1: Peripheral blood cell populations in SPF infant rhesus macaques

Age (weeks)	Mean Numbers / μl Blood ± SEM					Mean T Cell Numbers / μl Blood ± SEM		Mean Percentages of T Cells ± SEM	
	Neutrophils	Monocytes	Lymphocytes						
			Total	B Cells	T Cells				
						CD4$^+$T	CD8$^+$T	CD4$^+$T	CD8$^+$T
0	7294±916	487± 86	2687±267	289± 10	2336 ±265	1879±212	441±51	81.1 ± 1.2	18.9 ± 1.2
2	4928±650	339± 71	4123±288	1083±190	2886±226	2210±180	678±58	76.3 ± 1.1	23.7 ± 1.1
4	3826±489	236± 30	3897±246	1368±130	2328±60	1804±127	567±47	76.3 ± 1.2	23.7 ± 1.2
6	3484±523	222± 49	4889±317	1593± 80	2915±58	2193±197	712±61	75.2 ± 1.1	24.8 ± 1.1
8	2941±300	267± 48	4878±308	1912±165	2845±58	2076±172	753±63	73.2 ± 1.0	26.8 ± 1.0
10	3473±476	292± 67	5421±305	1890±143	3091±57	2194±168	830±55	71.9 ± 1.2	28.1 ± 1.2
12	2501±286	180± 21	5500±309	1975±135	3232±58	2341±173	934±77	71.6 ± 1.2	28.4 ± 1.2
16	2769±354	162± 22	5171±280	1559± 45	3128±61	2243±135	930±75	71.0 ± 1.3	29.0 ± 1.3
20	4008±592	246± 31	6100±358	2308±385	3739±61	2562±215	1150±99	68.9 ±1.4	31.1 ±1.4
24	3258±507	359± 86	5181±377	1917±228	3222±61	2223±230	934±89	69.7 ±1.2	30.3 ±1.2
28	2995±375	283± 64	5083±331	2018±241	2648±54	1969±158	805±63	70.5 ±1.4	29.5 ± 1.4
32	2721±445	226± 61	4746±272	1514±124	2584±53	1840±161	767±50	69.8 ± 1.4	30.2 ±1.4
36	2780±445	274± 70	4826±229	1348±235	2745±57	1933±139	801±55	70.0 ±1.3	30.0 ± 1.3
40	2028±190	268± 49	4838±283	1318±177	2796±59	1910±132	870±77	68.7 ±1.4	31.3 ±1.4
44	2649±546	257±104	5143±397	1841±425	3150±58	2148±222	975±95	69.1 ±1.3	30.9 ±1.3
48	2217±273	341±102	4665±291	1746±487	2937±63	1981±200	917±82	68.8 ± 1.3	31.2 ± 1.3
