# Supplementary material for: The interplay between immune maturation, age, chronic viral infection and environment
Source: Immun Ageing. 2015 May 9;12:3. doi: 10.1186/s12979-015-0030-3 (PMC4436863; doi:10.1186/s12979-015-0030-3)
Supplement: Additional file 2: — Memory T cell development in infant SPF-2 macaques. [file 12979_2015_30_MOESM2_ESM.pdf]

Table S2: Memory T cell development in infant SPF-2 macaques

Age (weeks)	CD4+T			CD8+T		
	T(N)[a]	T(CM)	T(E/EM)	T(N)	T(CM)	T(E/EM)
	Percent ± SEM [b]					
0	97.6±0.31	2.2±0.31	0.3±0.20	88.7±1.21	5.1±0.79	5.7±0.74
2	93.2±0.87	6.5±0.86	0.4±0.21	79.9±1.78	5.7±0.66	12.3±1.54
4	89.8±1.21	9.4±1.09	0.6±0.18	73.4±2.56	10.2±1.09	15.4±1.76
6	90.1±1.07	9.3±0.98	0.5±0.18	78.6±1.96	8.5±1.10	12.8±1.30
8	91.0±0.92	8.3±0.89	0.4±0.09	79.8±1.76	7.7±0.76	12.1±1.24
10	89.2±1.12	10.2±1.09	0.5±0.09	76.5±1.44	8.9±0.81	14.7±1.07
12	90.4±0.81	9.2±0.77	0.3±0.08	79.4±1.58	7.0±0.67	13.2±1.17
16	90.8±0.87	8.9±0.85	0.3±0.05	79.9±1.25	7.4±0.73	12.3±0.90
20	90.1±1.09	9.4±1.02	0.4±0.07	76.7±2.70	7.1±0.73	16.1±2.37
24	88.6±1.34	10.9±1.37	0.4±0.09	73.9±2.94	8.4±0.96	17.2±2.59
28	86.4±1.00	13.1±1.00	0.3±0.08	70.4±2.40	8.5±0.74	19.8±2.56
32	85.4±1.26	14.3±1.14	0.3±0.10	74.0±1.73	10.0±0.95	15.4±1.22
36	85.4±1.21	14.1±1.17	0.4±0.14	72.5±2.05	9.3±0.91	17.7±1.96
40	85.5±1.41	12.7±1.29	1.5±1.16	67.9±5.08	15.1±3.96	16.8±3.96
44	87.0±1.02	12.2±0.92	0.8±0.38	77.1±2.13	10.1±1.03	12.5±1.38
48	85.6±0.98	13.7±0.91	0.4±0.10	73.4±1.28	11.7±0.77	14.5±1.05

[a]CD28 and CD95 were used to define naïve ($CD28^+CD95^-$), central memory ($CD28^+CD95^+$), and effector/ effector memory T cells ($CD28^-CD95^+$).

[b] Values are expressed as percentages of $CD4^+$ or $CD8^+$T cells.
