# Supplementary material for: The interplay between immune maturation, age, chronic viral infection and environment
Source: Immun Ageing. 2015 May 9;12:3. doi: 10.1186/s12979-015-0030-3 (PMC4436863; doi:10.1186/s12979-015-0030-3)
Supplement: Additional file 3: — IFN-γ responses in infant T cells. [file 12979_2015_30_MOESM3_ESM.pdf]

Table S3. IFN-γ Responses in Infant T Cells

Age (weeks)	Animal No.	Number of SPF-2 Infant Macaques with Cytokine Responses							
		Percent of IFN-γ-producing $CD3^+CD4^+$T Cells				Percent of IFN-γ-producing $CD3^+CD8^+$T Cells			
		<0.015	≥0.015<0.1	≥0.1<1.0	≥1.0<10.0	<0.015	≥0.015<0.1	≥0.1<1.0	≥1.0<10.0
0	13 [a]	9 [b, c]	4	0	0	2	3	8	0
2	21	10	6	4	1	4	5	9	3
4	20	5	8	7	0	5	0	8	7
6	16	2	9	5	0	0	1	9	6
8	22	3	10	9	0	0	1	16	5
10	23	7	13	3	0	0	5	17	1
12	22	4	13	5	0	2	4	13	3
16	21	4	13	3	1	2	1	16	2
20	22	4	13	5	0	0	6	14	2
24	22	1	11	10	0	1	2	19	0
28	22	3	8	9	0	0	2	14	6
32	21	3	5	10	3	1	2	14	4
36	20	1	5	11	3	0	2	13	5
40	17	0	2	14	1	0	1	12	4
44	19	1	3	14	1	1	0	14	4
48	18	3	5	10	0	0	2	15	1

[a] total number of animals tested at this time point; [b] number of animals (out of the total animal number tested) who mounted IFN-γ specific $CD4^+$T cell responses within the specified range; [c] grey-shaded boxes indicate the response range for the majority of animals at any specific time point
